# Proceedings of the American Institute—Selected Extracts

**Published:** 1894-08

**Authors:** 


### PROCEEDINGS OF THE AMERICAN INSTITUTE—SELECTED EXTRACTS.

The jubilee convention of the American Institute of Homœopathy opened at the First Baptist Church, in Denver, Colorado, Thursday, June 14th, shortly after 3 o'clock. The church was comfortably filled with delegates and there were many spectators, consisting mostly of lady friends of the physicians. The edifice was handsomely decorated. The Stars and Stripes were entwined about the railing of the chancel and covered the front of the choir. The gallery was also smothered in American flags, while at regular intervals about the walls appeared cards bearing the names of the founders of the Institute. Palms and sweet-smelling flowers graced the speaker's stand. All the balustrade of the organ loft was draped with rich American flags. Two others of silk, with gold tassels, mounted, stood before the large columns on each side of the altar. The raised platform, upon which the officers were placed, was covered with flags. Pots of tropical plants ornamented the corners and the floor in front. A unique feature of the decoration was the long row of shields, each set between two small flags, and placed along the whole lower front of the balcony and in the rear of the stage. All except four of them were black, with white lettering, giving the names of the dead members of the convention held fifty years ago for the organization of the Institute of Homœopathy. The white shields, with black lettering, showed the names of the original members who are still living. In the middle of the balcony, directly in front of the platform, was a large portrait of Hahnemann, the founder of the homœopathic school.

### ON THE BLACK SHIELDS.

Stretching to the ends of the balcony on each side were black shields, inscribed with the following names: Walter Williamson, Eliphalet Clark, James M. Quinn, Harvey H. Cator, Peleg Clark, Edward Bayard, Luther Clark, Alonzo Ball, William Channing, John F. Gray, Albus Rea, George Lingen, Erastus

Humphreys, Gustavus M. Taft, Adolphus F. Haynet, Abraham D. Wilson, Stephen R. Kirby, George W. Swazey, Amos G. Hull, Joseph H. Pulte, Josiah F. Flagg, Phineas P. Wells, John Payne, Henry D. Paine, Dr. Spalding, Ralph A. Snow, George W. Cook, Henry E. Dunnell, John C. Gosewisch, Charles D. Williams, John Taylor, Charles Wild, Francis Sims, John A. McVickar, Horatio Robinson, Felix R. McManus, John Merrill, Abraham H. Okie, Henry F. Joslin, Eben Hale, William Wesselhœft, Charles J. Hempel, Adolphus Lippe, Constantine Hering.

These were the names on the white shields behind the platform: Joseph C. Boardman, Isaac Ward, Charles Neidhard, James Kitchen.

These shields, speaking silently the honor of the founders of the Institute, formed with the flags draped and tacked up everywhere a very effective scheme of decoration.

The opening session was devoted to business. President J. H. McClelland made his formal address, and was followed by the chairman of the Executive Committee, who made the committee's report. Other reports followed, and the regular order of business was taken up.

There were three candidates for the office of President—Dr. Comstock, of St. Louis, one of the oldest physicians in the Institute; Dr. Fisher, of Chicago, and Dr. Mitchell, of the same city.

President McClelland's address was short and pithy. He told the objects of the Institute and the reasons for the convention, and made a few pleasing remarks about Denver and its people. Then he mentioned the business that was to be transacted and retired. The presentation of reports followed.

PRESIDENT'S ADDRESS.

"In opening this jubilee meeting of the Institute of Homœopathy, I feel the honor which is mine. My aim has been and shall be to do my duties impartially. I shall not have much to say at this time. My annual address will be deferred till Friday evening. Still, I would now say a few things.

"This session is marked by unusual proceedings. It is the jubilee year of our organization. I would offer a few suggestions only. There is much less occasion than usual for modification of our by-laws. Distinctive duties have been applied to the office of General Secretary. I would recommend the restoration of a salary of $1,000 to the Secretary, because of the work he has to do. I am sure the Institute is now in his debt. Our body has doubled in numbers in late years, and with that increase comes an increase of labor. I would also recommend the creation of the office of Registrar, who shall have charge of registration, badges, etc.

"I wish also to call attention to the position occupied by the school in public affairs. Formerly we have been occupied in the close study of our profession. It is our first duty to heal the sick. But it has dawned upon us at last that there is a new science under the name of preventive medicine. It should be and is rapidly coming to be one of the chief duties of physicians to prevent disease as much as is possible. Sanitary science is coming to the front in these days of progress. There should be in all colleges courses of study in this science, and all medical study should embrace this science.

"I beg to call attention to this fact, too. Homœopaths are not now sufficiently recognized in public affairs. It is held that we constitute a sect in medicine. This idea should be eradicated. Homœopaths are thoroughly qualified as medical men, and I would suggest that this convention adopt a resolution to the effect that we claim our just share of public recognition as citizens and practitioners."

The President's address was referred to a committee, which Vice-President Fisher named, to consist of Drs. I. T. Talbot, William Tod Helmuth, and L. H. Willard.

### BUREAU OF MATERIA MEDICA.

Dr. Frank Kraft, chairman of the section, opened the proceedings by stating that a departure had been inaugurated in its work. Instead of reading the full papers, it was necessary to condense each on account of the great number received. Every professor of materia medica in the United States was represented, with one exception.

Dr. Timothy Field Allen, of New York, was the first speaker, who spoke on the potash salts in the materia medica. The speaker held that the drinking of alkaline waters was extremely harmful, and mentioned the waters of some of the much advertised Eastern springs as "simply devilish." The waters containing excess of potash were particularly dangerous. In the matter of tuberculosis patients, the speaker defined his reasons for sending them to Colorado. The tubercle germs, he said, were around us in all forms, and the most powerful disinfectant against them was pure blood.

Dr. A. L. Monroe, of Louisville, said that the name of the previous speaker, Dr. T. F. Allen, should be embalmed in the history of the Institute, along with Dr. Dake's, as most eminently advanced in the treatment of materia medica by therapeutics.

The matter was referred to a committee. The chair asked Dr. A. C. Cowperthwaite, of Chicago, to open the discussion on the remarks of Dr. Allen. He said that after hearing the remarks, he felt very much as though he wanted to take another course of materia medica. Other speakers were heard on the subject, and Dr. Allen closed with a few explanations as to his previous remarks. The section voted a rising vote of thanks to Dr. Allen, after which the section adjourned.

### BUREAU OF CLINICAL MEDICINE AND PATHOLOGY.

The second paper was on "Scarlet Fever," and was read by Dr. W. H. Hanchett, of Omaha. The speaker said that this disease was not as contagious as many others peculiar to children, but the peculiar germ belonging to this disease was peculiarly tenacious. It could be claimed that the disease had a specific principle or germ that could be transmitted in many ways, but fortunately many who are exposed are not susceptible to its ravages. A statement of the general symptoms showed it to be closely allied to diphtheria and it was probable the two acute diseases could run their course together. The homœopathic school had achieved bright laurels in the treatment of this disease. It has shown its great superiority over the allopathic treatment, the percentage of deaths being far less and the dangerous and disagreeable sequels which so often follow this disease less frequent. The speaker said he very much preferred the hot pack, believing that there was less danger in its use. It had been his custom for years to use fresh lard freely and frequently over the entire body, applying it as often as every hour during the intense fever. This allays the itching, irritation, and fever, often reducing temperature a degree by a single application and holding it down by its repeated application. Sponging is admissible if intelligently done. The diet should be light during the fever; nothing is better than good fresh milk, the patient being allowed water whenever desired in reasonable quantities. Many other forms of food are admissible, such as beef tea, arrow root, and various forms of gruel. Little solid food should be given a child during the first week of the fever. During the disease thorough disinfection should be employed. During the disease a carbolized spray should be frequently used in the sick room. After the patient has recovered thorough fumigation should be practiced, nothing being better than the common sulphur candle. "As homœopathic physicians we may well feel proud of the results obtained from our treatment of this disease. If it can be shown, as I believe it can, that the asylums of our country are filled with the deaf, dumb and blind from lack of skillful treatment and that there is a better way known to the followers of Hahnemann, may we not hope that in the future our efforts and success will be appreciated by a grateful public?"

Dr. Comstock, during the discussion, said that his method of treatment was to put the child in hot sheets and place cold compresses on the head, reducing the fever slowly. Whisky or champagne were recommended as proper stimulants. Dr. Skiles said he thought hot water was sufficient in nearly all cases, and that when the temperature rose to 104 the child was placed in hot packs until the fever was brought down to 101 degrees or less. Various other speakers spoke of cases which had come under their supervision. Dr. Coggswell held that nursing was quite as important as the medicine in diseases of this nature, and held that disinfectants were not necessary in any cases, the regular homœopathic remedies being sufficient. Other speakers claimed that disinfectants had been of great assistance in their practice.

Dr. Wiley said that in about four hundred cases he had wrapped the patient in cloths wet with a solution of bicarbonate of soda solution and the regular homœopathic remedies in addition, and in none were the results otherwise than favorable. Dr. H. C. Allen said the disinfectant was one of the humbugs of the other school, and that as a previous speaker had said, the homœopathic remedies were sufficient. He also held that one dose was sufficient in many cases; the patient did not have more than one exposure to the disease, and the Hahnemann theory did not demand more than that.

Dr. Timothy Field Allen said his practice was to disinfect the sheets, etc., but could not understand why some doctors wanted to disinfect the inside of the patient. "When it comes to pitting Homœopathy against hydropaths or antipyretics, I am a homœopath every time," he concluded.

Dr. Culver compared the speakers to a "tumbling box" in a foundry, and explained that the friction of the pieces in the box had made each one bright, though it made much noise. He was positive he was much brighter than when he first came into the hall.

Dr. Gordon said it was the habit of some to run perfectly wild when they got into certain lines, microbes, germs, or anything else. When he was in the army the young surgeons were trying to keep their knives bright by use, and they cut off everything in sight. But soon they got tamed down and were conservative in their practices, and the results were better. He deprecated the tendency of the young surgeons of to-day to do the same. He hoped these matters would not be lost sight of. Dr. Gordon's remarks were probably the most lively of the session. He was extremely emphatic and eloquent, and caused some merriment by his spread-eagle style of oratory.

Dr. Comstock, in replying to some of the remarks, said that the presence of albuminuria in scarlet fever cases was not necessarily fatal ; in fact, he had found it present in more than half the cases under his notice in greater or less extent. During desquamation he had the patient take a bath once a day and thoroughly anointed with vaseline twice each day.

Dr. Hanchett closed the discussion on his paper. Nearly all the points covered were agreed upon that much explanation was not necessary. He said : "I am in favor of disinfection of rooms and clothing as a preventive to the spread of the disease. The boards of health of nearly all cities require it. With due care in regard to this matter there is no danger of a physician attending lying-in cases at the same time scarlet fever cases are under his charge."

Dr. T. E. Roberts read a short but very instructive paper on the multiple stethoscope, the particular advantage of which is that the instrument permits four persons to listen to the heart action of the patient at the same time. An illustration of the workings of the instrument was given. The chairman announced that the instrument was not new, having used a similar instrument for thirteen years.

Dr. W. J. Martin, of Pittsburg, read a paper on "Gall Stones," and explained his method of treating this most painful disease. The homœopathic preparation of China, he said, was a most important drug for the relief of gall-stone colic by removing the cause. The treatment is commenced with the sixth dilution and gradually decreasing the dose. Hypodermic injections of Morphine may sometimes be necessary during the attack. Records of several cases were given and the use of China demonstrated in each.

Dr. Wright, of Buffalo, led the discussion on the paper. Dr. Thayer, of Boston, he claimed, introduced the treatment to the Institute. The Morphine was discarded by the originator, but the China was given more frequently. Dr. Thayer's practice was to give the sixth potency only. Other speakers said that Podophyllum, Calcarea carbonate and Leptandra were also useful in cases of this nature. The general opinion was that careful treatment with China or Cinchona was successful in all cases, and its use for a few months generally prevented a recurrence of the attack.

The chairman, Dr. Crawford, said he could not be convinced that either China, Calcarea carbonate or any other drug could be of any use whatever in what he considered purely a mechanical disease, and he would still hold on to Morphine.

"You cannot overcome that mechanical condition without something more than a dynamic remedy. It was a simple mechanical fact of a rough crystal about a quarter of an inch in diameter passing through a tube about the size of a goose quill lined with extremely sensitive nerves. The use of Olive oil is a mechanical means on account of a portion of the oil being emulsified and acting as a partial lubricant and solvent of the gall stones."

There were a number of other papers on the programme which were not read on account of the lack of time.

### PRESIDENT JAMES H. M'CLELLAND'S JUBILEE ADDRESS.

The evening session was attended by nearly all the delegates and their friends. The platform was occupied by the Institute officers and members of the local committee. Dr. Storke introduced the President, Dr. James H. McClelland, who delivered the Jubilee Address, as follows:

"Mr. President, Ladies, and Gentlemen:—We are assembled this evening amid anniversary scenes and memories. A jubilee year is upon us—the first in the history of the American Institute of Homœopathy, over which I now have the honor to preside. Nor can I fail to observe the significance of the place of our assembly—at the foot of those mighty mountains, whose steadfastness and grandeur shadow forth the stability and achievements of the truths we celebrate; and in this wonderful city, in whose energy and riches we read the first historical pages of the new and vast empire now rising in the centre of our continent. I congratulate you, and in so doing find the nature of my address suggested by the hour, associated as it is, with a splendid array of facts now spread before medical science; and by the place illustrating, as it does, the spirit which had borne thus far forward the lamp of knowledge and built upon eternal law this great superstructure of advanced medicine. We take pride in the past. We can look it in the eye and are ready to welcome those anniversaries with a good account of our stewardship.

"Of medicine, as of other sciences, it is true that the present is a display of truths hidden in the storehouses of the past. An historical parallel between the old and the new befits the occasion. We will confine ourselves within the limits of the last half century, which measures the life of our national body. Our inquiries will lead us to notice the status of medicine in general—a science made up of many parts, the crown and cap stone of which is that embodiment of truth in therapeutics—Homœopathy.

"In 1844 the great moving cause of reformation in an important branch of the healing art had vanished from the scene of his labors. Was it possible that the truths he espoused and elaborated should perish from the earth? That they did not is evidenced in the gathering together of a few faithful men who sought to perpetuate the doctrine of Hahnemann by forming an organization for the purpose. As individual labor must be necessarily limited these men gathered their energies into organic shape and the result was the American Institute of Homœopathy. The history and achievements of this great body have been ably set forth by my distinguished colleagues, Drs. J. P. Dake and I. T. Talbot, in their admirable historic addresses with which you have already been favored.

"The Hahnemann College, of Philadelphia, was the first in the new school, as the University of Pennsylvania was the first in the old, to extend hospitality to this important study. In Europe it had an honored place in all scientific curricula, particularly in Germany, in inorganic lines. In France liberal government patronage enabled it to reach even secondary grades, having obtained in 1840 a sure foothold in eighteen preparatory schools. In America it has made remarkable progress since

1865. In its highest branches it can now be studied where heretofore facilities were inadquate or wholly wanting. It has gone down into secondary institutions until there is not an academy or high school in the land but sends to medical schools matriculates with better qualifications than previous terms of admission ever required.

"A wide and radical change of sentiment pervades, I am pleased to say, the schools, the profession, and the country in the matter of medical instruction tending toward a higher standard of qualification.

"Half a century ago perhaps no branch of medical science had more accurate knowledge or within given scope yielded more comprehensive results than anatomy. During the second quarter of this century this study presented the strange anomaly of freedom from text book and instructor. Possibilities were supposed to be exhausted. The crudity of mechanical means limited investigation mostly to the skeleton. The first valuable contribution to English literature on the subject was in 1848, by Prof. Owen, who referred to the skeleton almost exclusively. In anatomy as a whole 1894 differs from 1840 mainly because of the microscope.

"Physiological science underlies surgery, anticipates pathology, co-operates with therapeutics, and is grounded upon anatomy, physics, and chemistry. For these reasons its development was early and has been continued. Modern physiology here, with improved microscope, evinces large thoughts and wider range. A world of phenomena, hitherto beyond its grasp, has been revealed by methods of induction, experiment, and classification. Electricity investigates the properties of nerve and muscle and localizes cerebral functions. The microscope explores intracellular processes. The bacillus has been dragged from its hiding place and made to tell the story of its felonious functions. The conception of the nutrition of the body has been revised and materially changed. However, although these sciences have fathomed many seas and scaled many heights, the horizon of fifty years seems but little nearer.

"Hygiene, from the fall of the Roman empire until the present century, had advanced into its second quarter. Sanitary science was relegated to limbo. A millennium of filth had met its judgment day in 1348, when the black death, during its prevalence, purged Europe of 25,000,000 of begrimed beings, who had been cursed in the name of godliness with an unreasonable opposition to the laws of health, preferring rather to live in filth, breathing foul air, and drinking polluted water. Scarcely a human being in Europe took a bath for one thousand years. Though disaster prepared the way for a better Europe, yet repeated visitations of the plague were necessary before preventive medicine with the trumpet to its lips obtained a hearing.

"Canada, Mexico, and the United States are soliciting the co-operation of the South American States for the redemption of the entire continent from the reign of epidemic diseases. Incalculable results, with greater to follow, have already accrued to efforts in our own land. Two years ago, at our Eastern ports, cholera was held at bay until it sank into the sea. This accursed heritage of barbarism and the Middle Ages, brought to our shores by the smitten hordes who would smite us, may be kept safely housed on the Ganges. The yellow fever is routed from the South. By destroying the excretory products of consumption, hygiene breaks the wand of that contagion which has stricken down one-seventh of those now numbered with the dead. The spread of animal diseases, which are rapidly emigrating from Europe to America, is being checked.

"No one greater instrumentality promises greater returns to society for the expenditure incurred, in saving of money, health, suffering, and life itself, than the sanitarium. In view of which fact it is a matter of regret and surprise that State governments in their dealings with them should exhibit such an extraordinary degree of parsimony.

"It is difficult to name the time when the step was taken which gave rise to bacteriology, which was destined to modify so much that had been accepted as pathology. It is said to have originated as a hypothesis in the year 1650 during a visitation of the plague by Athenius Kircher, a learned monk, since whose days it has at intervals risen for discussion.

"Before the day of Hahnemann practice matured into those conditions which were to justify in medicine a reformation analogous to that in the church in the sixteenth century. The want of a therapeutical system gave full scope to an individual nationalism. This was the condition that prevailed when Hahnemann awakened to a realization of the deplorable condition of medicine and began a systematic exposure of its fallacies. Hahnemann lived to witness Homœopathy secure with a firm footing by right of conquest and the popular will. In 1840 he and his followers achieved their remarkable conquest over Asiatic cholera—an auspicious historical inauguration of the new *régime*. Homœopathy had stepped forth as the shepherd lad of Bethlehem against the brusque Philistian giant and at the time the American Institute was organized there were probably not more than one hundred physicians in the United States who had espoused its cause. The *Materia Medica Pura* and Happ's *Archives* were the only guides, with sixty drugs, and of these but twenty were proven. As late as 1836 no scientific treatises upon the new science had appeared in the English language.

"Fifty years have now passed—a period sufficiently long for history to begin to formulate its verdict in the perpetuated controversy. The two great schools have largely developed in their respective lines. Both confess to the same mission—to heal the sick. One is almost exclusively occupied with the researches of physiology and pathology—the other emphasizes therapeutics and materia medica, while making the totality of medical science tributary to the cure of human ills. It is historically certain that there has been no advance in the old school of practice from within itself. That a radical change has transpired in that school cannot be controverted. The average practitioner is at sea and the pharmacist, quick to take advantage of the situation, makes the remedies and foists them upon him as factories furnish the mechanic with material ready made. Science is no longer a necessity. It is in vain that chemistry and the microscope yield their triumphs unless the patient be cured.

"Hahnemann defines disease as a disturbance of vital force. He defined medicine as a knowledge of disease. His definition is conclusive and must end controversy. Riots of prejudice but evidence their verity. It is indisputable that it has the right conception of therapeutics.

"Before the clinical use of a drug it asks its properties, its pharmaceutical purity and present energy, and then what phenomena will follow its introduction into the human organism, what effect upon structure and function. To this principle every school of medicine must ultimately come. The old school beats the devil around the bush by experimenting upon healthy animals and reptiles. But provings upon the lower animals are not reliable. Healthy human beings alone with all their differences and idiosyncrasies can be fit subjects for exact experiment.

"Men and brethren, what glorious memories and priceless blessings are associated with the name and achievements of this great discoverer in medical science—the savior of lives. His followers have built hospitals and colleges, and are numbered by thousands and tens of thousands.

"The American Institute by its duly constituted committee is preparing to erect in the national capital a fitting memorial in granite and bronze to that illustrious name. A work which will be carried to completion and in which all who bear that name as followers should have a share. Let us 'highly resolve' that by our tributes there shall rise as the crowning expression of our gratitude a memorial of such grandeur that his name and work shall remain the heritage of the ages."

The address by the President was received by the audience with marked appreciation, and upon its conclusion the speaker took his seat amid much applause.

### TECHNIQUE OF WOUND DRESSING.

Dr. Willard's paper on "Technique of Wound Dressings in Modern Surgery." Thirty or forty years ago operations which are now performed without hesitation were considered not only fatal but foolish to undertake on account of the mortality. A very potent factor in this advance of surgery is the careful attention now given to the smallest details during the operation, and in the course of the after treatment.

Dr. Willard proceeded to say that after an operation, such as amputation of a limb, wounds should be dry before dressed, and then dressed without drainage. In amputations it was once considered necessary to insert a drainage tube between the flaps in order to give egress to serious oozing.

The speaker did not favor the location of an open tube in a wound, which he characterized as a highway for admission of infectious germs. Besides its removal necessitated an early and otherwise unnecessary redressing. He also said that drainage may be avoided in many instances in compound fractures by thoroughly cleansing the wound, trimming away all torn and ragged tissues, relieving tension, checking all hemorrhage, and serious oozing, and by fixed plaster of Paris dressing, with provision for fenestra, if necessary.

Poultices were said by Dr. Willard to be one of the last remaining relics of surgical barbarism. It is a good rule never to use a poultice to an open wound. It was yet the custom with some practitioners to fly to poultices for relief of pain and tension. Such poultices form a suitable ground for the propagation of germs. How much better, on the incipiency of such symptoms, is an early incision, which relieves tension, evacuates the forming pus, and checks further progress of the inflammation.

Dr. McDonald, of Washington, said Dr. Willard had omitted one of the most important points, early incision for suppuration. He favored Dr. Willard's position on tubes for drainage, and thought the iodoformed gauge tube the best for the purpose.

Dr. Walton, of Cincinnati, strongly advocated hot water, absorbent cotton, gutta-percha, and oiled paper as substitutes for the old-fashioned poultice.

Dr. Willard read Dr. Packard's paper on "The Repair of Denuded Areas by Skin Grafting." The special advantages of this method of repair are the rapidity with which it is effected, and that the resulting cicatrices do not contract.

Dr. Packard's paper read in part:

Skin grafting consists in the transplantation of small or large portions of integument from one part of the body to another.

The suggestion of a possibility of the continued vitality of a piece of skin tissue, which had once been separated from its original site, and reapplied thereto, or to some distant site prepared for it, was suggested long years ago, through the custom which has prevailed in India of punishing criminals by slicing off the nose. If the culprit or his friends could but secure the portion cut away, and it be reapplied to its original site within a short time, in many cases it would become adherent.

The same experience has followed accidental lopping off of the fingers. Under favorable circumstances such severed digits, if carefully readjusted to their original site, will promptly become adherent, and perfect repair follow.

Medical history shows that this possibility of skin transplantation was not utilized until toward the middle of the present century.

The transference of skin flaps by stages, such as the reconstruction of the nose by borrowing a flap from the forearm (Tallacotion method), or forehead (Indian method), should not properly be classed under skin grafting, since such flaps of skin are allowed to remain attached to their original site, by a pedicle of sufficient size to preserve their vitality until adhesions have formed to the new place of attachment and circulation established from a new source.

The first attempt at actual skin grafting was made by Riverdin about 1870. His method consisted in the transference of minute cuttings, scarcely larger than the head of a pin, from some eligible portion of the body, to the denuded area. These minute grafts he placed in rows and in case of large areas to be covered, hundreds, and, perhaps, thousands of grafts were applied.

This method marked a great step in advance, and proved a great aid in hastening the cicatrization of some cases of ulcers, and large areas denuded of skin through burns, etc. Those who have practiced this method in the past will recall certain discouraging features identified with it, viz., the many grafts, even however carefully placed, that were lost through failure to adhere; many that seemed to be swallowed up by profuse granulations; and in many cases the entire failure of any grafts to take.

This method of Riverdin's was, however, but a single step in the evolution of the now well-established

### THIERSCH'S METHOD.

In the author's opinion, this method of repair of large denuded areas serves to rank with the greatest achievements of surgery.

We are all familiar with the long, tedious months of waiting for denuded areas, such as result from burns, to cover over. We also know of the hideous deformities which result from cicatricial contraction, following the natural healing of burns. Who has not seen everted eyelids, distorted mouths, or contracted limbs as a result of nature's method of repair? Thiersch's method offers a certain and rapid mode of repair in these painful cases without cicatricial contraction, and with a far less hideous scar than is effected by nature.

Let us glance for a moment at the process which nature follows, if left to herself. An area over which the whole thickness of the integument has been destroyed must be covered in, if at all, by the generation and regeneration of epithelium from the healthy integument around the borders of the wound. While such epithelium is gradually creeping toward the centre, granulation tissue is piling up all over the wound surface. This rapidly becomes changed into a kind of connective tissue, and thus layer after layer of this reparative tissue becomes formed until months and perhaps years have passed, the epithelium from the surrounding borders has gradually crept over and the wound is healed. The granulation connective tissue beneath has, in the meantime, gradually contracted, dependent in a large degree upon the area of the body thus affected, and the extent of the original wound, with corresponding deformity.

What we may ask is, in brief, this beneficent discovery which obviates this slow process of repair and prevents deformity? It is the transference of strips of integument, as large and as thin as can be conveniently cut from some hairless portion of the body, preferably the anterior aspect of the thigh, and sufficient in area to completely cover the denuded surface.

The whole process must be conducted on the strictest aseptic lines. Strong antiseptics such as might endanger the vitality of the delicate strips of skin, or the cells of the area prepared to receive them, must not be allowed to come in contact with the tissues.

DETAILED TECHNIQUE.

Preparation of Denuded Area.—An inexorable condition for success is that the area to be grafted shall be in a good state of vitality. The grafts will take upon a freshly wounded surface, also upon a healthy granulating surface.

A wound covered with unhealthy granulations may be curetted until sound and resisting tissue is reached, and the grafts applied directly to this, or, if a covering of healthy granulations can be induced, curetting is unnecessary. It is all important that the surface should not be in a state of putrefactive suppuration. A perfectly clean matrix is a primary essential.

Salt Solution.—The free use of a solution of common salt (.6 of 1 per cent.) approximating the salinity of blood is a feature of Thiersch's method. The last item in every step of the process is the free douching of the field of operation and its accessories. If a chemical antiseptic, such as Bichloride of Mercury, be used to disinfect the denuded area, that from which the grafts are to be taken, or the operator's hands, the last step is to wash it off with salt solution. All sponges, dressings, and instruments are first made aseptic by heat or chemical antiseptics, and lastly bathed in the salt solution.

Cutting of the Grafts.—The anterior surface of the thigh is the most convenient portion of the body from which to take the grafts, and in the majority of individuals it is practically hairless. The area from which they are to be taken is prepared the night previous to the operation, by scrubbing with three changes of soap and water, shaved, packed in 1 : 3,000 sublimate gauze, which is allowed to remain until shortly before the operation. It is then removed, the surface washed with the salt solution and packed with gauze wrung in the same.

Instruments and Dressings.—The best apparatus for cutting the grafts, in my experience, has been Mixter's graft cutter, which consists of a fenestrated steel plate, a knife and a squegee. In addition to this will be needed scissors, and probes for shaping and adjusting the grafts.

Strips of gutta-percha, three-fourths of an inch wide, should be cut long enough to reach across the wound, and lap on both sides. They are prepared by washing in soap and water, then soaked over night in 1 : 1,000 sublimate solution, and, lastly, washed in salt solution. An abundance of the salt solution must be at hand in basins and pails at the temperature of the human body, in which to frequently immerse instruments and the operator's hands, and to sop over the field of operation.

### OPERATION.

Anæsthetize the patient; make the area to be grafted clean and smooth by scraping away any superabundant or unhealthy granulations; bevel off any prominent ridges of skin about the margins; pack with pledgets of aseptic gauze wet in salt solution and bind tightly with a bandage, or have an assistant exert pressure to stop hemorrhage. All capillary oozing must have ceased before the grafts are applied.

Expose the anterior surface of the thigh, which has been prepared; lay the fenestrated steel plate over the surface from which the first strip of skin is to be cut. Sharp pins will be found on the under surface of the steel plate, which will serve to fix it in position while the grafts are being cut. On pressing the steel plate well down upon the surface of the thigh, it will be seen that the skin bulges up through the fenestrated opening. With the knife in the right hand and the squegee in the left, cut the graft by resting the knife flatwise on the steel plate and, with a sawing movement, make the thinnest possible section. This can be accurately gauged by causing the roller of the squegee to be pressed down upon the steel plate, immediately preceding the edge of the knife.

The graft, as it is cut, will slide over the knife and remain lying upon its original site. In transferring, it must be handled carefully or it will roll and become unmanageable. Its adjustment is best effected by laying upon it a piece of tissue paper wet in the salt solution, and then gently lifting both together; the graft will be found adherent to the tissue.

The surface to be grafted, if all oozing has now ceased, receives the graft, which can be shaped, with the aid of scissors, to inequalities in outline, by simply cutting through the graft, with the tissue upon which it lies. It is then placed upon the denuded area, and the edge of the graft held in position while the tissue is stripped off. After the whole area has been covered in this way, the strips of gutta-percha tissue are laid across, each slightly overlapping, as with clapboards. The whole is then enveloped in many layers of gauze wet in the salt solution, with a layer of cotton batting over this, and the whole held snugly in place by a bandage.

The dressings are kept moist by occasional application of the salt solution. The dressings are changed every second or third day. By the tenth day the grafts have become firmly united in their place and assumed a pinkish color; the outermost layer of epidermal cells has peeled off and the area has healed. No further treatment is necessary beyond daily bathing and the application of Calendula or Boracic acid cerate.

### TRANSPLANTING A LARGE FLAP.

A case illustrative of a large flap of skin from the flank to the forearm was then quoted as follows:

Mr. E., age forty-six, presented himself with the following history: When a lad six years old, he was one day standing before an old-fashioned fire, when, apparently from the effect of a recently contracted cold, he suddenly fainted, and fell headlong into the flames. When rescued the right arm was found very severely burned. The skin was totally destroyed over the anterior portion, from the shoulder to the base of the fingers. In six months from the date of the mishap the wound had healed. All went well with the youth until four years later, when a small, reddish elevated patch appeared in the cicatrice near the flexure of the elbow. This gradually encroached upon the surrounding cicatricial tissue, and for fifteen years, or until the patient was twenty-five years of age, maintained an unbroken surface.

About this time it began to ulcerate near the flexure of the elbow, accompanied by a sero-purulent discharge. The ulceration and discharge gradually increased. From year to year but little progress could be noticed, but through a period of ten years the growth and accompanying ulceration had attained a size approximating the palm of the hand. For eighteen years it went on in this slowly progressing way, and discharged profusely. This continued for two years, which brings it to the date when the patient first came to my notice. At the time I first examined him the right arm was flexed at an angle of about sixty degrees; the index finger was dislocated from the long-continued contraction of the cicatrice, and drawn backward so that the base of the first phalanx stood upon the dorsum of the metacarpal bone, and the finger was flexed at the remaining articulations to the last degree. The whole hand was also drawn backward slightly, by the extensor action of the cicatricial bands. From about five inches above the flexure of the elbow to eight inches below the site of the cicatrice was occupied by an enormous keloid, widely ulcerated, and with the edges raised and everted. At its widest part the growth measured three and three-eighths inches.

The patient was a farmer and all these years had followed arduously all the laborious work ordinarily falling upon one of that occupation. Winters he had worked for weeks at a time in the logging camp, wielding the axe as effectively as his companions, and summers he swung the scythe and pitched hay with equal vigor. It seems a plausible conclusion that the extreme tension which such severe labor constantly brought upon the contracted cicatrix at least greatly aggravated the growth.

After heroically bearing the discomfort attendant upon such a state of matters for all these years he at last gave up, believing that nothing remained but to have the arm amputated, and he consulted me with that end in view.

A careful examination of the arm showed a sound and vigorous condition of all the muscles; the patch of skin only seemed involved, and with the exception of the presence of the annoying ulcer and accompanying discharge, and the flexure of the arm at the elbow, the limb was strong and serviceable. Under the circumstances I could but strongly urge against amputation.

The possibility of the removal of the diseased mass by free dissection and the repair of the gap by the transplantation of a flap of skin from some distant portion of the body, presented itself. After duly considering this plan it was determined to attempt it. In pursuance therewith the patient was duly etherized, the field of operation thoroughly disinfected, and the whole mass rapidly dissected off. The skin and subcutaneous connectives were cleanly removed down to the muscular fascia. A muslin pattern was then cut exactly the shape of the wound, and its form marked out on the right flank in such a position that when the arm was brought down to the side of the body the prospective flap could be laid over on the arm, and thereby cover the wound. The flap was cut about one inch larger than the pattern, to allow for contraction. It was rapidly dissected up and left attached the whole length of the inner border—that is, the border nearer the umbilicus. The arm was then brought into position, and the flap laid over the wound. It covered it completely, and the edges were fastened by continuous silk sutures all around. The arm was fastened to the side by long, broad bands of adhesive straps, and the patient put in bed. Warm compresses were kept upon the flap for the first twenty-four hours. By this time the circulation seemed well established, and union appeared to be taking place along the united edges. During the second twenty-four hours the flap became considerably over-suffused with blood. It looked like skin which had been subjected to just sufficient heat to produce redness without vesication. During the third twenty-four hours a line of œdema appeared along the edge of the flap farthest removed from the pedicle. This œdema rapidly spread until the larger part of the flap was involved. Shortly after the first appearance of the œdema a leaden-colored border appeared along the edge where the œdema was first seen. It was evident that sloughing of a portion of the flap at least must occur. The œdematous swelling of the flap lifted it considerably above the level of the surrounding skin, thereby separating the edges, which had apparently united, and tearing out the sutures. Sloughing progressed slowly during the next few days, and the necrotic tissue was trimmed away with the scissors as fast as its vitality was lost. Such shreds as were cut away were quite firmly united to the bottom of the wound, showing that adhesions had quickly formed between the inner raw surface of the flap and the muscular fascia. On the eighth day the flap was separated from its original site in the flank, and the arm set free. The flap very quickly changed color, after detachment, from the almost livid appearance which it had presented to an almost bleachy whiteness. It seemed firmly attached to its new location, the œdema rapidly diminished, and the sloughing quickly ceased. The separation of the edges of the flap and the sloughing left quite a broad margin along the outer side uncovered. As soon as healthy granulations had sprung up skin grafts were thickly set.

The method pursued in transplanting the grafts was essentially the same as laid down in text-books, viz., the thorough disinfection of an area of healthy skin, the picking-up of a point of integument on a needle, and snipping off minute bits of the epidermis with sharp scissors. These bits were placed in rows across a selected area, covered with isinglass plaster, and allowed to remain undisturbed for three days. This method of holding the grafts in place proved unsatisfactory, for the reason that the plaster very soon became softened by the discharge, the edges rolled up, and the grafts washed away. Hardly two per cent. of the grafts so treated took. It must be evident that if much aid was to be gained through skin grafting, a different method of fixing the grafts in position must be devised. To that end, strips of organdy muslin, a wide-meshed, thin fabric, were used in place of the plaster straps. They were drawn tightly over across the line of grafts, and the ends which lapped over on to the sound skin were plastered down with collodion. This device worked to a charm. The wide meshes of the muslin permitted free exit for the discharge, and at the same time held the grafts securely in place. From this time on, the healing progressed without interruption; the new islands of epidermis spread rapidly and coalesced.

As soon as the arm was freed from the body, the large wound in the flank, caused by the removal of the flap, demanded as constant attention as the arm. This rapidly filled up to a level with the surrounding skin with granulation, and skin-grafting was resorted to after the same method as already described.

The operation was performed on the 8th of May, and the patient returned to his home in New Hampshire on the 10th of the following August with arm and side soundly healed.

At the time of the separation of the flap on the eighth day, considerable trepidation was felt lest death of the whole flap should follow. Subsequent developments seem to indicate that an earlier division even might have been better.

From the second day, there was certainly an over-supply of blood in the flap, as indicated by the œdema. It is quite possible that the capillary blood-vessels all over the base of the flap rapidly united with those of the denuded surface of the arm beneath. It is also quite conceivable that the lymphatics were much slower than the blood-vessels in uniting, and thereby failed to furnish exit for the increased amount of lymph poured into the tissue of the flap, from the double source of blood supply, viz., the original attachment of the flap, and the new adhesions beneath.

The method of holding the grafts in place with the strips of organdy muslin and collodion is, I think, an original one, and, from the success attending its use here, is worthy of further trial.

### COMMENTS ON THE PAPER.

Dr. McDonald, of Washington, agreed with the points in Dr. Packard's paper and was in accord with the practice adopted by him.

Dr. Walton, Cincinnati, contended that the first essential to success in skin plantation was that the soil should be adequately and properly prepared. You might as well boil wheat and corn before planting as expect to be successful in skin grafting without proper preparation. Failure in this operation was in the main to be attributed to lack of reparation.

Dr. P. C. Majumdar, Calcutta, India, sent a paper on the "Progress of Surgery in its Relation to Homœopathy." It was mainly a high tribute to Dr. Helmuth and other prominent homœopathic surgeons.

At 6.15 o'clock the session adjourned to next Monday morning.

At 8 o'clock this evening the memorial service in honor of the deceased members of the Institute will be held in the First Baptist Church. This morning many of the visitors will make the trip around "the Loop," some more will make a journey to Colorado Springs and go to Pike's Peak over the cog road. For these excursions special rates have been made by the railroads at the solicitation of the local branch of the Institute. In addition visits have been arranged to the mint, the smelters and other local objects of interest.

### THE SECTION OF PÆDOLOGY. DR. VAN BAUN ON SCURVY IN INFANCY—OTHER PAPERS.

The session of the pædological section was mainly devoted to the criticism of diseases from a purely scientific standpoint. Dr. William W. Van Baun is President of the section and Dr. Charles A. Gale, of Rutland, Vt., Secretary. The essays read were "Hahnemann's Doctrine of Psora in the Treatment of Diseases of Children," by Dr. William Bœricke, of San Francisco; "Prevention of Deformities," by Dr. Millie J. Chapman, of Pittsburg; "Barlow's Disease," by Dr. Deschere, of New York city; "The Paralysis of Diphtheria," by Dr. O. E. Janney, of Baltimore, Md.; "The Sexual System and Procreation," by Dr. J. C. Nottingham, of Bay City, Mich.; "The Feeding of Sick Children," by Dr. B. H. R. Sleight, of Newark,

N. J., and "Cholera Infantum," by Dr. C. H. Thomas, of Cambridge, Mass.

Dr. William W. Van Baun, of Philadelphia, Editor of the *Hahnemannian Monthly*, read a paper on "Scurvy in Infancy and Childhood," which has recently awakened so much interest with the specialist in children. Said he: "There is no case on record without a clearly defined history of impoverished blood due to the lack of those products furnished by fresh food, milk, and fruits. As a causative factor artificial or proprietary food takes a prominent and distinguished place, although anything given in the line of nourishment overtaxing the infant's digestive system, coupled with a deficient supply of fresh animal or vegetable products, will tend to develop the cachexia. Meat juices given alone for a long time will also cause scurvy. So many infants are fed on patent baby food, which insufficiently nourishes, that the children of the wealthy more frequently develop scurvy than those whose parents are poverty stricken and are forced to resort to table scraps during the early period of their lives. The treatment is largely dietetic, calling for the free use of fresh food and drink, together with a plentiful supply of grape or orange juice."

Dr. Charles A. Gale discussed tubercular meningitis, emphasizing the necessity of early recognition of the disease for treatment of any kind to be of any use. All treatment must be in the premonitory stage. He showed that under homœopathic treatment this disease is amenable to treatment, and that many children who would otherwise die young under treatment go on to perfect health.

